# Use of T-SPOT.TB for the diagnosis of unconventional pleural tuberculosis is superior to ADA in high prevalence areas: a prospective analysis of 601 cases

**DOI:** 10.1186/s12879-020-05676-2

**Published:** 2021-01-04

**Authors:** Xinting Yang, Jing Zhang, Qingtao Liang, Liping Pan, Hongfei Duan, Yang Yang, Hua Li, Chao Guo, Qi Sun, Hongyan Jia, Boping Du, Rongrong Wei, Aiying Xing, Zongde Zhang, Xiaoyou Chen

**Affiliations:** 1 National Clinical Laboratory on Tuberculosis, Beijing Key Laboratory for Drug Resistant Tuberculosis Research, Beijing Chest Hospital, Capital Medical University, Beijing Tuberculosis and Thoracic Tumor Institute, Beijing, China; 2 Department of Tuberculosis, Beijing Chest Hospital, Capital Medical University, Beijing Tuberculosis and Thoracic Tumor Institute, Beiguan St #9, Beijing, 101149 China

**Keywords:** Interferon gamma release assay (IGRA), Adenosine deaminase (ADA), Tuberculosis, Pleural fluid

## Abstract

**Background:**

Tuberculous pleural effusion (TPE) is the most common extrapulmonary manifestation and may have lasting effect on lung function. However conventional diagnostic tests for TPE register multiple limitations. This study estimates diagnostic efficacy of the interferon gamma release assay (IGRA: T-SPOT.TB) in TPE patients of different characteristics.

**Methods:**

We performed a prospective, single-centre study including all suspected pleural effusion patients consecutively enrolled from June 2015 to October 2018. Through receiver operating characteristic (ROC) curves, technical cut-offs and the utility of T-SPOT on pleural fluid (PF) were determined and analysed. Logistic regression analysis was performed to obtain the independent risk factors for TPE, and evaluated the performance of the T-SPOT assay stratified by risk factors in comparison to ADA.

**Results:**

A total of 601 individuals were consecutively recruited. The maximum spot-forming cells (SFCs) of early secretory antigenic target-6 (ESAT-6) and culture filtrate protein-10 (CFP-10) in the PF T-SPOT assay had the best diagnostic efficiency in our study, which was equal to ADA (0.885 vs 0.887, *P* = 0.957) and superior to peripheral blood (PB), with a sensitivity of 83.0% and a specificity of 83.1% (The cut-off value was 466 SFCs/10^6^ mononuclear cells). Among the TPE patients with low ADA (< 40 IU/L), the sensitivity and specificity of PF T-SPOT were still 87.9 and 90.5%, respectively. The utility of ADA was negatively related to increasing age, but the PF T-SPOT test had a steady performance at all ages. Age (< 45 yrs.; odds ratio (OR) = 5.61, 95% confidence interval (CI) 3.59–8.78; *P* < 0.001), gender (male; OR = 2.68, 95% CI 1.75–2.88; *P* < 0.001) and body mass index (BMI) (< 22; OR = 1.93, 95% CI 1.30–2.88; *P* = 0.001) were independently associated with the risk of TB by multivariate logistic regression analysis. Notably, when stratified by risk factor, the sensitivity of PF T-SPOT was superior to the sensitivity for ADA (76.5% vs. 23.5%, *P* = 0.016) and had noninferior specificity (84.4% vs. 96.9%, *P* = 0.370).

**Conclusions:**

In conclusion, the PF T-SPOT assay can effectively discriminate TPE patients whose ADA is lower than 40 IU/L and is superior to ADA in unconventional TPE patients (age ≥ 45 yrs., female or BMI ≥ 22). The PF T-SPOT assay is an excellent choice to supplement ADA to diagnose TPE.

## Background

Tuberculous pleural effusion (TPE), one of the most common forms of extrapulmonary TB, has a presentation spectrum from fully absorbed benign to complicated pleural thickening and even serious complications such as empyema and fibrothorax, which may have a lasting effect on lung function. Early and effective diagnosis could minimize hospital days and maximize quality of life. At present, the most direct evidence for *Mycobacterium tuberculosis (MTB)* infection is aetiology [1], which has suboptimal sensitivity. Therefore, patients whose pleural effusion is characterized by lymphocytic exudates [2] combined with high ADA levels are frequently diagnosed as having TPE by clinicians, resulting in high prevalence.

Adenosine deaminase (ADA), an enzyme produced from lymphocytes that is involved in purine metabolism, has been used in the diagnosis TPE for a long time due to its excellent sensitivity and specificity [3, 4]. However, recent studies have shown that patients with empyema, malignancy, or rheumatoid pleurisy can also have high ADA levels [5], and negative results in older TPE patients [6] and fluctuation were obviously affected by the patient profile and local tuberculosis (TB) prevalence [7].

The interferon gamma release assay (IGRA) is a commercially available cost-effective assay that detects changes in interferon gamma (IFN-γ) caused by *MTB* infection. The WHO guidelines [8] rejected the recommendation of the IGRA for differentiating active TB, especially in high-burden countries, but the guidelines published by the European Centre for Disease Prevention and Control (ECDC) [9] proposed that the IGRA could contribute supplementary information for patients who test negative for acid-fast bacilli (AFB) and *MTB* culture in sputum. In recent years, an increased number of studies have researched the utility of the IGRA to diagnose TPE. A recent meta-analysis [10] assessing the performance of the IGRA for diagnosing TPE exhibited satisfactory outcomes (PPV = 82%, NPV = 87%), but the results were heterogeneous (I^2^ = 92.0–82.5%), suggesting that the results are still controversial and polarizing. Theoretically, tuberculosis antigen-specific responses by IGRA should provide a discriminatory value superior to non-specific inflammatory biomarkers (e.g., unstimulated IFN-γ or ADA), but this is not the case. We boldly presume that the superiority of the IGRA could be concealed by discrepancies in population characteristics, causing inconsistencies between studies.

Currently, there are two commercial kits for IGRA; one is an enzyme-linked immunosorbent assay (ELISA), which detects the IFN-γ in the whole blood and is represented by the QuantiFERON-TB Gold test [9], which was approved by the FDA in 2004; the other test is the enzyme-linked immune-spot assay (ELISpot), which detects IFN-γ released by mononuclear cells isolated from whole blood under the stimulation of specific antigen, represented by the T-SPOT assay [9] developed by Oxford University. Both methods have similar principles, but they are slightly different in detection technology and concrete operation. To further to clarify the diagnostic role of the IGRA for TPE in patients with a high TB burden, this prospective study was conducted to investigate the utility of the IGRA assay (T-SPOT.TB) in the discrimination of TPE and to compare the difference in potency with ADA in TPE subjects with different characteristics.

## Methods

### Participant population and study procedure

A prospective study was performed at Beijing Chest Hospital, Capital Medical University, from June 2015 to October 2018, in which all suspected pleural effusion (PE) patients were enrolled consecutively. The enrolled patients met the following criteria: (1) age ≥ 14 years; (2) presentation with PE on chest ultrasonic examinations; and (3) tolerated thoracic puncture and had more than 100 ml pleural effusion. The exclusion criteria were as follows: (1) HIV positive; (2) a history of immunodeficiency, autoimmune disease or use of immunosuppressive drugs; and (3) previous anti-tuberculosis treatment for more than 2 weeks.

Clinical samples (including sputum, peripheral blood (PB) and pleural fluid (PF)) from all participants were processed for diagnostic purposes after obtaining written informed consent (P.S. The consent of the participants below age 18 years were obtained from their guardian); tests included routine clinical biochemical testing for each PF sample, which contains total protein, glucose, lactate dehydrogenase (LDH) and ADA, and smear microscopy, culture and Gene-Xpert for each sputum and PF sample. All participants’ clinical data were extracted by the investigators, and the treatment process and discharge diagnoses were tracked. All participants were followed up for 12 months to verify the final diagnosis, and patients with negative outcomes after anti-TB treatment in the last 12 months were deemed indeterminate diagnoses.

### Clinical categories of pleurisy

Patients were divided into three groups according to the composite reference standard (CRS), which was composed of clinical, laboratory, and radiological examinations and follow-up data of diagnostic treatment. (1) Bacteriologically confirmed TPE was represented by the isolation of *MTB* in PE, sputum or pleural tissue by culture, microscopy or Gene-Xpert, or a pleural biopsy that demonstrated caseating granulomas. (2) Probable TPE lacked bacteriological confirmation, but all patients were treated empirically for TB based on clinical suspicion (e.g., typical clinical symptoms, remarkable radiological imaging and positive outcome of anti-TB treatment during follow-up). (3) Non-TPE indicated cases were diagnosed definitively as other diseases, such as malignancy or empyema (non-tuberculous disease).

### ADA measurement

ADA activity was determined colorimetrically at 37°C using a commercial kit (Adenosine Deaminase Assay Kit; Beijing Strong Bio-technologies, Beijing, China) according to the peroxidase assay [11]. One unit of ADA was defined as the amount of enzyme that generated one micromole of inosine from adenosine per minute at 37°C. The results were expressed in international units per litre of PE (IU/L).

### T-SPOT.TB in PF and PB

The PB (4 mL) and PF (45 mL) samples collected from each participant were tested within 6 h. The PB samples were diluted 1-fold and centrifuged at 900 g for 20 min, and the PF samples were centrifuged at 500 g for 10 min. The supernatant of both samples was discarded for T-SPOT.TB testing.

The T-SPOT.TB assay was conducted following the manufacturer’s instructions (Oxford Immunotec Ltd., Oxford, UK), which were identical for both the PB and PF samples. The pellets were resuspended in 8 mL of AIM-V medium (GIBCO, Rockville, MD, USA). Briefly, mononuclear cells (MCs) were separated using FicolleHypaque, washed, resuspended, and counted. Empty wells were used as negative controls, the T lymphocyte mitogen phytohemagglutinin was used as a positive control, and the ESAT-6 and CFP-10 peptides were in separate wells. Isolated peripheral blood mononuclear cells (PBMCs) and pleural fluid mononuclear cells (PFMCs) were added to the wells (2.5 × 10^5^ cells per well) that were precoated with a monoclonal antibody against IFN-γ and incubated at 37 °C for 16 to 20 h. The spot-forming cells (SFCs) were read using an automated enzyme-linked immunosorbent spot (ELISPOT) reader (CTL-ImmunoSpotS5 Versa Analyser). A test was considered valid when the positive control > 20 SFCs/10^6^ mononuclear cells and the negative control < 6 SFCs/10^6^ mononuclear cells. The final SFCs of ESAT-6 or CFP-10 were defined as ESAT-6 or CFP-10 SFCs minus negative control SFCs. The Max SFCs of the T-SPOT assay were defined as the larger of the final ESAT-6 and CFP-10 SFCs.

### Diagnosis

#### Smear, acid-fast bacilli (AFB) and mycobacterial culture

Specimens including sputum and PF (5 mL) were prepared for direct smear and stained with auramine and examined by light-emitting diode microscopy. The smear was read and interpreted in accordance with the WHO guidelines [12]. The sputum and PF (5 mL) were preprocessed using N-acetyl-L-cysteine and sodium hydroxide (NALC-NaOH) and centrifuged, and the supernatant was discarded. The resuspended pellet was transferred to solid Lowenstein-Jensen medium (Encode Medical Engineering Co., Ltd., China) and liquid medium and subjected to culture in a mycobacterial growth indicator tube (MGIT) 960 system (Becton, Dickinson and Company, USA). The presence of the *MTB* complex in any medium represented positive MPT64 antigen testing. The positive events and time were recorded.

#### Gene-Xpert

The Gene-Xpert test was performed according to the manufacturer’s instructions (Cepheid, Sunnyvale, CA, USA). Briefly, the specimen (including sputum and concentrated PF) and sample reagent were fully premixed at room temperature. The final 2 ml mixture was collected and transferred to the cartridge and loaded into the automatic Gene-Xpert instrument. Duplicate testing was performed on samples with an invalid result.

### Statistical analysis

Data were analysed using IBM SPSS 25.0 (SPSS Inc. Chicago, IL, USA) GraphPad Prism 8.2.1 (GraphPad Software, Inc. La Jolla, USA). Quantitative variables are presented as the mean ± standard deviation (SD) or median (interquartile range (IQR)), and categorical variables are presented as frequencies (percentages). To identify differences between two independent groups, the chi-square test was used to detect differences between categorical variables, and the Mann-Whitney U test and unpaired *t*-test were used for continuous data in non-normal or normal distributions, respectively. A result was considered statistically significant when the *P*-value was < 0.05.

Receiver operating characteristic (ROC) curves were plotted to evaluate the diagnostic performance of ADA and T-SPOT.TB, obtaining the optimal cut-off value and calculating the corresponding areas under the ROC curve (AUCs). The sensitivity, specificity, positive predictive value (PPV), negative predictive value (NPV), positive likelihood ratio (+LR), negative likelihood ratio (−LR), diagnostic odds ratio (DOR) and accuracy were calculated.

Predictors that were related to TPE by a predetermined *P*-value of 0.10 or less were selected and used in a multivariable logistic regression model (except symptoms). Stepwise backward selection using *P* < 0.10 was used to derive the model. Multicollinearity was assessed, and variables contributing to the best fit of the final model, or most related and widely available in our setting, were retained in the final model. For application of the model, a bioclinical score chart was derived using the adjusted OR value of the predictors [13]. Overall research was completed in keeping with the Standards for Reporting of Diagnostic Accuracy (STARD) template [14].

## Results

### Participant characteristics

**Fig. 1:**
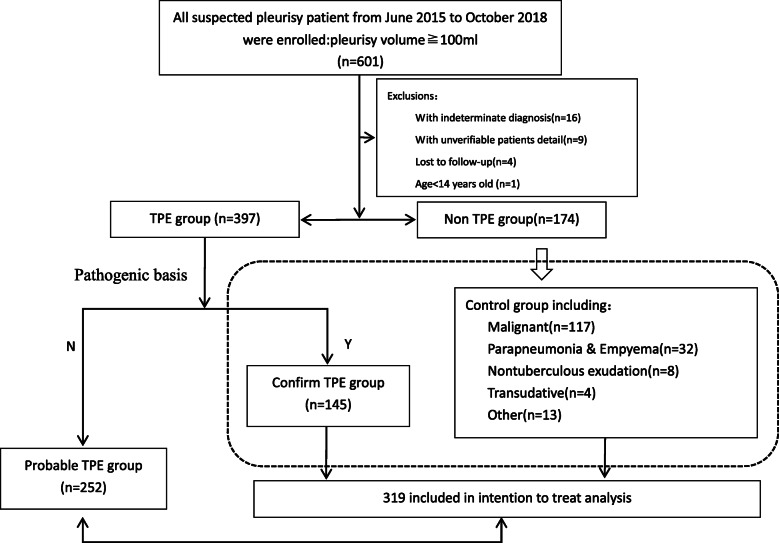
Study design. Numbers in parenthesis refer to the patients submitted to the correspondent diagnostic group. Dotted box contains all TPE patients (Confirmed & probable TPE groups)

From June 2015 to October 2018, a total of 601 suspected PE patients were enrolled, and patients with indeterminate diagnoses (*n* = 16) and unverifiable patient details (*n* = 9) and those who were age < 14 years old (*n* = 1) and lost to follow-up (*n* = 4) were excluded. According to the CRS, 145 patients had confirmed TPE, 252 patients had clinically diagnosed probable TPE, and 174 patients had non-TPE (Fig. 1). Among the 174 non-TPE patients, 117 (67.2%) were diagnosed with malignant pleural effusion, 32 (18.4%) with parapneumonia or empyema (non-tuberculous), 8 (4.6%) with exudation effusion (non-tuberculous), 4 (2.3%) with transudate effusion and 13 (7.5%) with other conditions (Fig. 1), which was in line with the current epidemiology of PE.

In the confirmed TPE group, the tests of sputum for AFB, culture, Gene-Xpert were positive in 27.6, 60.5 and 64.6% of patients, respectively, while PF was positive in only 2.2, 37.4 and 25.4% of patients, respectively, and a higher total detection rate in sputum (68.3% vs. 46.9%) showed that obtaining direct proof of *MTB* infection from PF is more difficult than from sputum (Table 1).

**Table 1:** Laboratory bacterial-positive percentage of diagnosis of TPE

Positive TB test	Confirm TPE (***n*** = 145)
Sputum	
Smear, AFB positive	35/127
Culture positive for MTB	72/119
Gene-xpert positive	73/113
Total positive tests	99/145
Pleural fluid	
Smear, AFB positive	3/137
Culture positive for MTB	52/139
Gene-xpert positive	36/142
Total positive tests	68/145

Data are presented as frequency (percentage)

*AFB* Acid-fast bacilli, *MTB Mycobacterium tuberculosis*, *TB* tuberculosis, *TPE* tuberculous pleural effusion

### Clinical, demographic and biochemical data

The demographic and clinical characteristics of all participants are summarized in Table 2. The patients in the TPE group were younger than those in the non-TPE group (42.15 ± 19.78 vs. 57.59 ± 15.36, *P* < 0.001), and there were more male subjects in the TPE group than the non-TPE group (75.3%, 299/397 vs. 59.2%, 103/174, *P* < 0.001) and males were predominant in unilateral PE (83.9%, 333/397). All features were in accordance with the clinical patterns of TPE [15]. According to BMI in Table 2, The patients in the TPE group was significantly thinner than the non-TPE group’s (21.70 ± 4.24 vs. 23.23 ± 3.37, *P* < 0.001). Patients with TPE more frequently presented with fever (74.8% vs. 39.7%, *P* < 0.001) but had less chest tightness (58.6% vs. 71.8%, *p* = 0.013) than patients without TPE. The patients with probable TPE inferred by clinicians had more obvious clinical symptoms relating to TB infection, such as more night sweats (21.4% vs. 10.3%, *P* = 0.009), more weight loss (34.1% vs. 23.6%, *P* = 0.042) and less haemoptysis (l.2% vs. 7.5%, *P* < 0.001). However, there were no significant differences in various characteristics between the confirmed and probable TPE groups (*P* > 0.05).

**Table 2:** Descriptive characteristics of participants (*N* = 571)

	Non-TPE (***n*** = 174)	TPE patients (***n*** = 397)				*P value
		Confirm TPE (***n*** = 145)	^***%***^***P*** value	Probable TPE (***n*** = 252)	^***§***^***P*** value	
Age, ys (Mean ± SD) ± sd) sdSD)	57.59 ± 15.36	45.00 ± 20.71	**< 0.001**^**#**^	40.5 ± 19.07	**< 0.001**^**#**^	**< 0.001**^**#**^
Gender(%)male	103 (59.2)	119 (82.1)	**< 0.001**	180 (71.4)	**0.009**	**< 0.001**
BMI (kg/m^2^)	23.23 ± 3.37	21.40 ± 7.43	**0.004**¶	21.88 ± 3.30	**< 0.001**¶	**< 0.001**¶
Onset time (day)	35 (20–90)	30 (14–60)	**0.033**^**#**^	21.50 (13.65–45.71)	**< 0.001**^**#**^	**< 0.001**^**#**^
Smoking						
None	107 (61.5)	91 (62.8)	0.817	164 (65.1)	0.450	0.532
Years of smoking	30.79 (20.25–41.73)	21.67 (7.67–33.33)	0.259^#^	13.67 (8.53–28.64)	**0.038**^**#**^	0.050^#^
Previous anti-TB treatment	31 (17.8)	44 (30.3)	**0.009**^#^	36 (14.3)	0.325	0.516^#^
**Symptoms**						
Fever	69 (39.7)	117 (80.7)	**< 0.001**	180 (71.4)	**< 0.001**	**< 0.001**
Cough	131 (75.3)	120 (82.8)	0.105	191 (75.8)	0.905	0.422
Chest pain	76 (43.7)	73 (50.3)	0.235	126 (50.0)	0.199	0.156
Chest tightness	125 (71.8)	85 (58.6)	**0.013**	190 (75.4)	0.411	0.537
Night sweat	18 (10.3)	22 (15.2)	0.195	54 (21.4)	**0.003**	**0.009**
Weight loss	41 (23.6)	41 (28.3)	0.338	86 (34.1)	**0.019**	**0.042**
Hemoptysis	13 (7.5)	3 (2.1)	0.057	3 (1.2)	**0.002**	**< 0.001**
**Effusion site**						
Left	66 (37.9)	56 (38.6)	0.900	93 (36.9)	0.830	0.928
Right	69 (39.7)	65 (44.8)	0.351	119 (47.2)	0.122	0.138
Bilateral	39 (22.4)	24 (16.6)	0.190	40 (15.9)	0.088	0.072
**Pleural effusion volume**						
Small	72 (41.6)	59 (40.7)	0.867	106 (42.1)	0.927	0.990
Moderate	52 (30.1)	42 (29.0)	0.832	66 (28.0)	0.382	0.486
Massive	49 (28.3)	44 (30.3)	0.693	81 (32.1)	0.410	0.451
Total protein(g/L)	34.36 ± 18.68	37.63 ± 19.57	**0.003**^**#**^	43.47 ± 14.56	**< 0.001**^**#**^	**< 0.001**^**#**^
Glucose (mmol/L)	5.81 ± 2.92	4.50 ± 2.36	**< 0.001**^**#**^	5.04 ± 2.17	**< 0.001**^**#**^	**< 0.001**^**#**^
LDH(U/L)	256 (158.25–482)	425 (276–704)	**< 0.001**^**#**^	377.5 (240.5–580.75)	**< 0.001**^**#**^	**< 0.001**^**#**^
ADA (IU/L)	11.8 (8.25–18.65)	50 (37.85–62.15)	**< 0.001**^**#**^	45 (31.875–57.9)	**< 0.001**^**#**^	**< 0.001**^**#**^

Data are presented as mean ± standard deviation (SD), median (interquartile ranges) or frequency (percentage)

*TPE* tuberculous pleural effusion, *BMI* body mass index, *TB* tuberculosis, *LDH* lactate dehydrogenase, *ADA* adenosine deaminase

^%^Comparisons were performed between confirm- and non- TPE group using chi-square test and ^#^Mann–Whitney U test and ¶unpaired t-test

^§^Comparisons were performed between non- and probable- TPE group using chi-square test and ^#^Mann–Whitney U test and ¶unpaired t-test

*Comparisons were performed between non TPE and TPE group using chi-square test and ^#^Mann–Whitney U test and ¶unpaired t-test

Through multivariate logistic regression analysis, age (< 45 yrs.; OR = 5.61, 95% confidence interval (CI) 3.59–8.78; *P* < 0.001), gender (male; OR = 2.68, 95% CI 1.75–2.88; *P* < 0.001) and BMI (< 22; OR = 1.93, 95% CI 1.30–2.88; *P* = 0.001), were included in the final model and were independently associated with the risk of TB (Table 3).

**Table 3:** Logistic regression analysis of risk factors for TPE

	Adjusted OR(95%CI)	***P*** value
**Gender**		
Male	2.68 (1.75–4.11)	< 0.001
Female	1.00	
**BMI**		
< 22	1.93 (1.30–2.88)	0.001
≧22	1.00	
**Age**		
< 45y	5.61 (3.59–8.78)	< 0.001
≧45y	1.00	

*BMI* body mass index, *OR* odds radio

### Diagnostic utility of the T-SPOT.TB assay for PB and PF

**Fig. 2:**
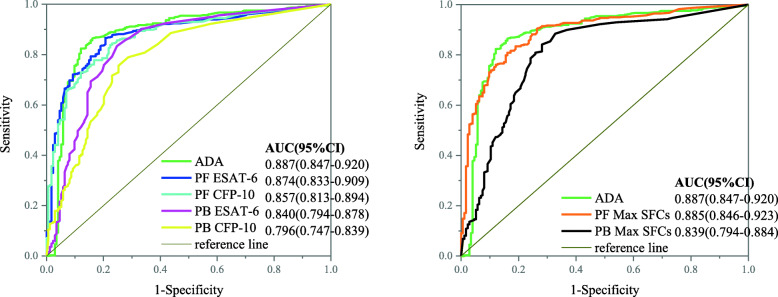
The receiver operating characteristic (ROC) curves of adenosine deaminase (ADA) and the T-SPOT assay for diagnosing tuberculous pleural effusion (TPE) in the pleural fluid (PF) or peripheral blood (PB). Samples were obtained from enrolled participants that included 145 patients with confirmed TPE and 174 with non-TPE. SFCs, spot-forming cells; Max, the larger of ESAT-6 and CFP-10

As shown in Fig. 2, the final ESAT-6 and CFP-10 and Max SFCs for PB and PF were affirmed to discriminate TPE from non-TPE, and no significant differences were observed between confirmed and probable TPE. In Table 4, we show the cut-off value of PB derived from the ROC analysis between the confirmed TPE group and the non-TPE group (Fig. 2), which is extremely close to the positive cut-off value (24 SFCs/10^6^ mononuclear cells) provided by the manufacturer. Overall, when we used the same cut-off value of 22 SFCs/10^6^ mononuclear cells, the performance of ESAT-6 was slightly better than that of CFP-10 in PB, with an AUC of 0.840 vs 0.796 (*P* = 0.055), as well as a sensitivity of 82.1% vs 75.2% (*P* = 0.123) and a specificity of 75.3% vs 77.9% (*P* = 0.847). However, when considering Max SFCs (cut-off value =22 SFCs/10^6^ mononuclear cells), the AUC, sensitivity and specificity were 0.839 (95% CI 0.794–0.884), 90.3 and 67.2%, respectively, which were no better than those of ESAT-6 (*P* = 0.954) (Table 4).

**Table 4:** Diagnostic utility of T-SPOT.TB (PF & PB) and ADA for diagnosing TPE

Variables	Cut-off value ^**a**^	Sensitivity%	Specificity%	PPV%	NPV%	+LR	-LR	DOR	AUC	95%CI
**Peripheral blood (PB)**										
ESAT-6	24^#^	77.9	77.0	73.9	80.7	3.39	0.29	11.80		
	22¶	82.1	75.3	73.5	83.4	3.32	0.24	13.98	0.840	0.794–0.878
CFP-10	24^#^	72.4	74.7	70.5	76.5	2.86	0.37	7.75		
	22¶	75.2	74.7	71.2	78.3	2.97	0.33	8.95	0.796	0.747–0.839
Max SFCs	24^#^	86.9	69.0	70.0	86.3	2.80	0.19	14.77		
	22¶	90.3	67.2	69.7	89.3	2.75	0.14	19.07	0.839	0.794–0.884
**Pleural fluid (PF)**										
ESAT-6	170¶	86.9	78.2	76.8	87.7	3.99	0.17	23.80	0.874	0.833–0.909
CFP-10	142¶	85.5	73.6	72.9	85.9	3.24	0.20	16.44	0.857	0.813–0.894
Max SFCs	466¶	81.4	82.8	79.7	84.2	4.73	0.22	21.07	0.885	0.846–0.923
ADA	22.4¶	89.0	83.9	82.2	90.1	5.53	0.13	42.16	0.887	0.847–0.920
	40	71.0	93.1	89.6	79.4	10.29	0.31	33.03		

*ADA* adenosine deaminase, *TPE* tuberculous pleural effusion, *NPV* negative predictive value, *PPV* positive predictive value, *+LR* positive likelihood ratio, *−LR* negative likelihood ratio, *DOR* diagnostic odds ratio, *SFCs* spot-forming cells, *Max* the larger of ESAT-6 and CFP-10, *AUC* the are under curve

^#^: manufacturer derived cut-off point for PB; ¶: AUC derived cut-off point

^a^ Values are expressed as IU/L for ADA, and as SFCs/106 mononuclear cells for T-SPOT

As expected, the performance of the PF T-SPOT assay was distinctly improved compared to the PB T-SPOT assay. ESAT-6- and CFP-10-specific cells were more highly concentrated in PF than in PB, with median ratios of 12.13 (IQR 3–29.4) and 9.30 (IQR 1.22–30.15), respectively, in the confirmed TPE group and median ratios of 11.87 (IQR 3.96–35.15) and 10.60 (IQR 2.63–32.21), respectively, in the probable TPE group, and no significance was observed between any groups. Based on the ROC analysis, the optimal cut-off point was 170 for PF ESAT-6, which produced a sensitivity of 86.9% and specificity of 78.2%, and the optimal cut-off point was 142 for PF CFP-10, which produced a sensitivity of 85.5% and specificity of 73.6%. Max SFCs in PF exhibited the best diagnostic efficiency, which was equal to ADA (0.885 vs 0.887, *P* = 0.957), with a sensitivity of 83.0% and a specificity of 83.1%, corresponding to a cut-off value of 466 SFCs/10^6^ mononuclear cells (Fig. 2, Table 4).

### Comparison of the diagnostic utility of ADA and the T-SPOT.TB assay stratified by bioclinical score

The median ADA levels in the non-TPE, confirmed TPE and probable TPE groups were 11.8 IU/L (IQR 8.25–18.65), 50 IU/L (IQR 37.85–62.15), and 45 IU/L (IQR 31.875–57.9), respectively (Table 2), confirming that a low ADA level was satisfactory in excluding TPE, but the ADA level in the probable TPE group was slightly lower than that in the confirmed TPE group (*P* = 0.055).

Each participant was grouped by scoring the logistic regression coefficient [16]. As shown in Table 5, when the score = 11 and the three risk factors were met (< 45 yrs., male and BMI < 22), the performance of PF T-SPOT was noninferior to ADA; compared to the stable utility of the PF T-SPOT assay, the sensitivity of ADA was positively related to score decline, while specificity was negatively related, suggesting that the PF T-SPOT assay performed better than ADA in unconventional patients, especially when the score = 0 (representing female patients whose age was more than 45 yrs. and BMI ≥ 22). The PF T-SPOT assay had a noninferior specificity (84.4% vs. 96.9%, *P* = 0.370) and a superior sensitivity (76.5% vs. 23.5%, *P* = 0.016) compared to ADA.

**Table 5:** Performance outcomes of a bioclinical score for ADA and T.SPOT

Score (non: TPE)	Assay (cut-off value ^a^)	Sensitivity	Specificity	PPV	NPV	Accuracy	§X^2^	§***P*** value	¶X^2^	¶***P*** value
	ADA(40)	23.5	96.9	80.0	70.5	71.4	5.82	**0.016**	0.80	0.370
**0** **(32:17)**	PB T.SPOT(24)	76.5	62.5	52.0	83.3	67.3				
	PF T.SPOT(466)	76.5	84.4	72.2	87.1	81.6				
**2**	ADA(40)	51.0	94.4	92.9	57.1	68.7	21.42	**< 0.001**	7.26	**0.006**
	PB T.SPOT(24)	79.4	64.5	76.4	68.3	73.3				
**/3/5** **(107:155)**	PF T.SPOT(466)	76.1	80.4	84.9	69.9	77.9				
**6/8/9**	ADA(40)	73.2	86.7	95.9	43.3	75.8	10.26	**0.001**	0.14	0.705
**(30:127)**	PB T.SPOT(24)	93.7	93.3	98.3	77.8	93.6				
	PF T.SPOT(466)	89.8	90.0	97.4	67.5	89.8				
	ADA(40)	81.6	80.0	98.8	18.2	81.6	0.04	0.840	0	> 0.999
**11** **(5:98)**	PB T.SPOT(24)	85.7	60.0	97.7	17.6	84.5				
	PF T.SPOT(466)	80.6	80.0	98.8	17.4	79.0				

*ADA* adenosine deaminase, *BMI* body mass index, *TPE* tuberculous pleural effusion, *NPV* negative predictive value, *PPV* positive predictive value

Age < 45 yrs., male and BMI < 22 scored 6, 3, 2 points, respectively

§Comparisons were performed for sensitivity between ADA and PF T.SPOT using chi-square test

¶ Comparisons were performed for specificity between ADA and PF T.SPOT using chi-square test

^a^ Values are expressed as IU/L for ADA, and as SFCs/10^6^ mononuclear cells for T-SPOT.Data are presented as percentage

### Comparison of the diagnostic utility of ADA and T-SPOT.TB stratified by age

**Fig. 3:**
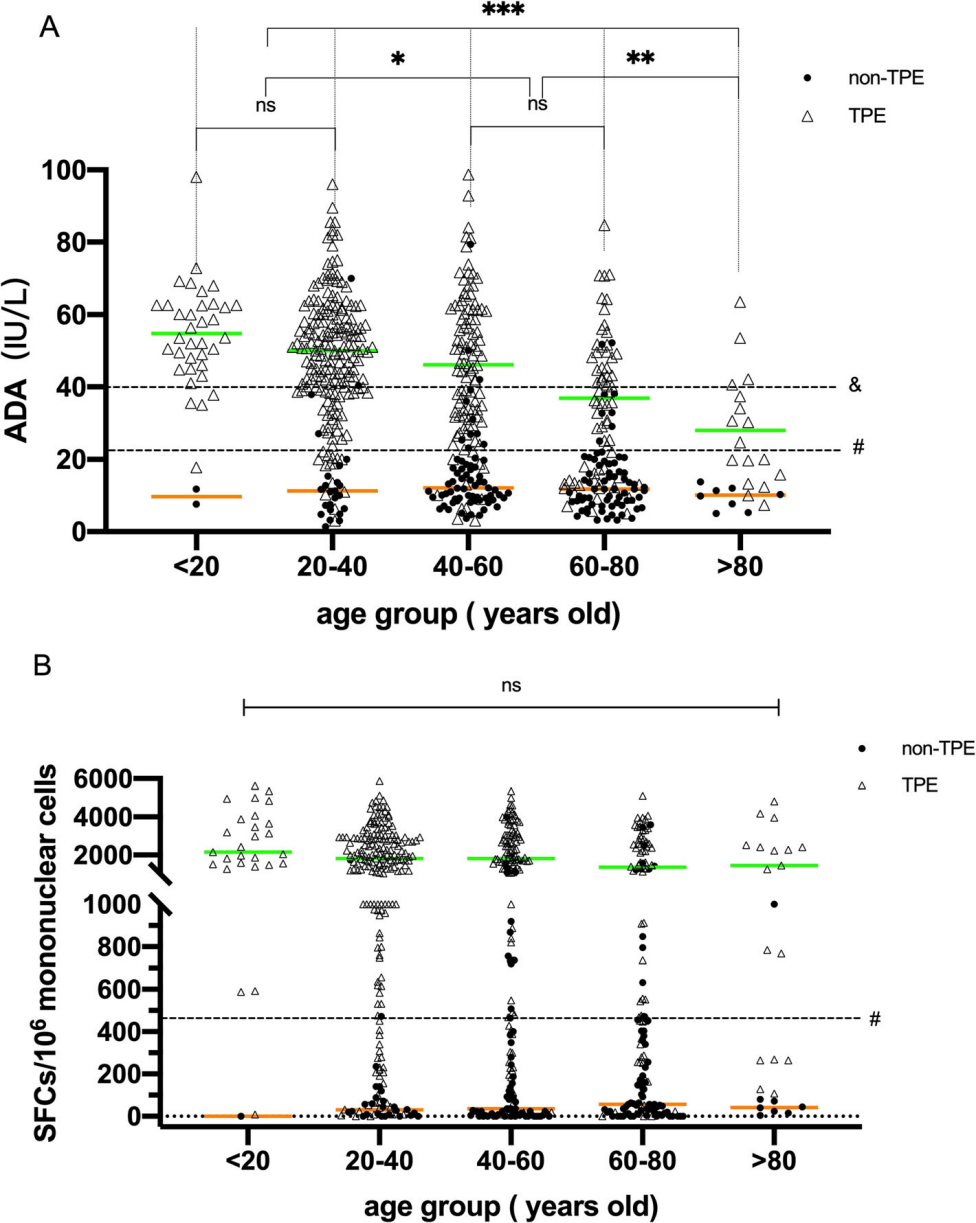
Comparison of the performance of adenosine deaminase (ADA) and the T-SPOT assay in the pleural fluid (PF) stratified by age. **a** Comparison of median ADA levels between different age groups. **b** Comparison of median spot-forming cells (SFCs) in the PF T-SPOT assay between different age groups. For group comparison, the Kruskal-Wallis test was used. ns, not significant. *p < 0.05,**p < 0.01,***p < 0.005; & represents internationally recognized cut-off (40 IU/L), and # represents the cutoff (22.4 IU/L for ADA, 466 SFCs/10^6^ mononuclear cells for PF T-SPOT) derived from the receiver operating characteristic (ROC) curves. The green and orange lines represent the median PF Max SFCs in the TPE and non-TPE groups, respectively

In Fig. 3a, the scatter plot shows that the distribution of ADA levels in the TPE group shifted downwards (*P* < 0.05) from age 40 years and above, especially the median ADA level in patients over 60 years old, which was lower than the clinical diagnostic point (40 IU/L), indicating that the ADA activity level was significantly negatively correlated with increasing age (*P* < 0.001); notably, the performance of the PF T-SPOT assay at different age stages was steady, with no significant differences among all groups (*P* = 0.604) (Fig. 3b). The above results showed that the performance of the T-SPOT.TB assay was superior to ADA in older patients.

### Diagnostic utility of the T-SPOT.TB assay in patients with indeterminate ADA (ranging from 20 to 40)

**Fig. 4:**
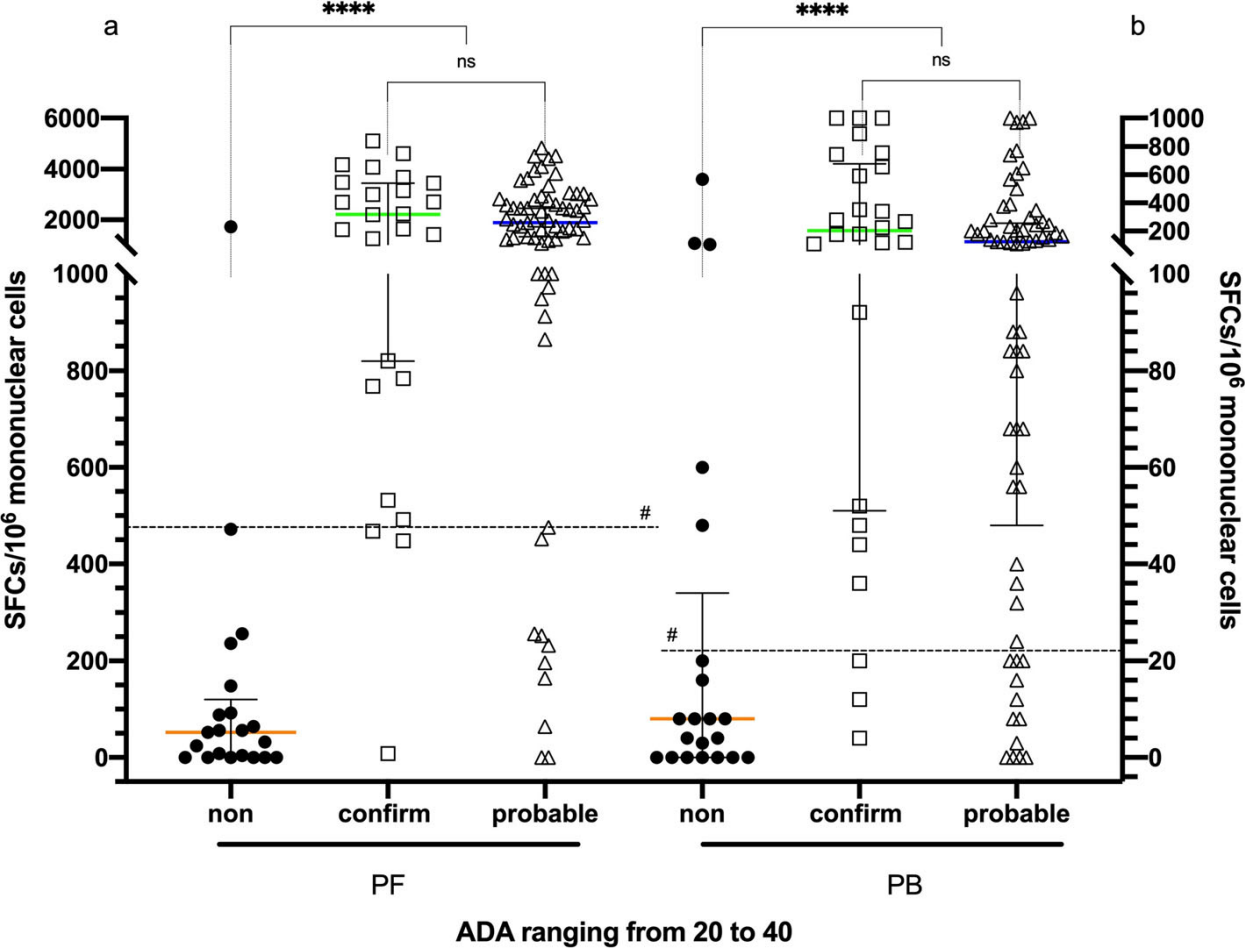
Diagnostic accuracy of the T-SPOT assay distinguishing tuberculous pleural effusion (TPE) patients with low adenosine deaminase (ADA) [ranging from 20 to 40]. **a** The T-SPOT assay showed a high sensitivity of 87.5% and a specificity of 90.5% in pleural fluid (PF). **b** The T-SPOT assay showed a relatively high sensitivity of 83.5% and a specificity of 76.2% in peripheral blood (PB). Comparison of spot-forming cells (SFCs) using the T-SPOT assay in PF and in PB between the confirmed TPE, probable TPE and non-TPE groups. For group comparison, the Mann-Whitney test was used. ns, not significant. ****p < 0.001; # represents the cut-off (22 SFCs/10^6^ mononuclear cells for PB T-SPOT, 466 SFCs/10^6^ mononuclear cells for PF T-SPOT) derived from the receiver operating characteristic (ROC) curves. The orange, green and blue lines represent the median PF Max SFCs in the non-TPE, confirmed TPE, and probable TPE groups, respectively

In our study, the cut-off value of ADA derived from ROC analysis was 22.4 IU/L, which had higher sensitivity (89.0%); conversely, when an ADA value of more than 40 IU/L was recognized as positive (international recommendations), it produced a higher specificity (93.1%). Therefore, we had 112 patients (19.6%) with ADA values ranging from 20 to 40 IU/L (21 in non-TPE, 26 in confirmed TPE, 65 in probable TPE),which were designated as ADA indeterminate. In Fig. 4, the scatter plot shows the diagnostic utility of the PF T-SPOT assay in the indeterminate analysis. The sensitivity, specificity, PPV and NPV were 87.9, 90.5, 97.6 and 63.3%, respectively. The Youden index was 0.784, and the sensitivity, specificity, PPV and NPV of the PB T-SPOT assay were 83.5, 76.2, 93.8 and 51.6%, respectively. The Youden index was 0.597. These data indicated that the T-SPOT.TB assay could discriminate TPE patients with an ADA value ranging from 20 to 40 IU/L, and the utility of the T-SPOT assay in PF was superior to that in PB.

## Discussion

To the best of our knowledge, although there are several publications that evaluate the utility of the T-SPOT assay for the diagnosis of TPE [9, 10], the majority of studies recruited a small sample population (*n* < 100) for evaluation, and the results were conflicting. Our study provided the largest cohort (TPE:non-TPE = 397:145) to date, confirming the value of the T-SPOT assay for the diagnosis of TPE with high confidence and providing specific reference suggestions for the clinical application of the T-SPOT assay.

In this study, all suspected PE participants had been consecutively, unselectively enrolled, and all TPE patients had a definite diagnosis by bacteriological confirmation or positive outcome for anti-TB therapy, which highly reflects the demographic epidemiological characteristics of tuberculosis-prone areas. The recruited samples ranged from adolescents to elderly patients over 90 years of age, confirming again that the TPE patients had the clinical characteristics of younger males, combined with higher ADA activity and higher T-SPOT response for PF and PB. Comprehensively, the specificity of the T-SPOT assay for PF was much higher with approximate sensitivity than that for PB, which is similar to other studies [17]. Moreover, different from the low incidence areas where the cut-off of PF T-SPOT was equal to PB T-SPOT’s [18, 19], our research affirmed that the diagnostic cut-off obtained from PB was not the optimal for PF in the high prevalence areas, and PFs’ cut-off was much higher than PB,which was in line with the expectation of another high-burden settings, for example, 300 SFCs/10^6^ mononuclear cells in Korea [20].

ADA, the most common biomarker for the diagnosis of TB pleurisy, has a value of more than 40 IU/L in lymphocyte-dominated PE and carries a PPV of 98% in high TB endemic regions [3, 4], while a retrospective analysis on ADA in 1637 subjects obtained an NPV of 100% with less than 15.0 IU/L [21]. In our study, we found that recognizing > 40 IU/L as the sole indicator of TPE may not be the most suitable approach, as it obtained high specificity while sacrificing sensitivity; 35.5% (141/397) of TPE patients had an ADA level that was lower than 40 IU/L in this study, 29.8% of which had a definite aetiological basis. In addition, the utility of a cutoff of 22.4 IU/L derived from ROC analysis was better than 40 IU/L (Youden index 0.729 vs. 0.641), this situation was similar to the publication of Santos et al. [22]. However, when comprehensively considering the non-specific elevation of ADA levels caused by non-tuberculosis inflammatory PE [19] (particularly complicated parapneumonic effusions and empyemas) and lymphomas, patients whose ADA was more than 20 IU/L and less than 40 IU/L were classified as indeterminate ADA. Conversely, the PF T-SPOT assay showed excellent diagnostic utility between ADA indeterminate groups, and its accuracy was higher than 90.2%, and we predict that the result of the PF T-SPOT assay could be a considerable indicator for highly suspected TPE patients with indeterminate ADA.

Many previous studies [23, 24] indicated that the performance of diagnosing TPE for patients aged more than 45 yrs. is still unknown. A recent study found that only 4.65% of elderly TPE subjects had levels over 40 IU/L [25]. Our research fully demonstrated this phenomenon simultaneously. In addition, we observed that the unclear boundary influenced by age for ADA was opportunely concentrated in the ADA indeterminate groups. Among the patients in the ADA indeterminate group, 85.7% (18/21), 73.7% (19/26) and 47.7% (31/65) of patients aged more than 45 yrs. were in the non-TPE, confirmed TPE and probable TPE groups, respectively, while the superior performance of the PF T-SPOT assay in distinguishing between ADA indeterminate groups may be explained by its steady performance in all age groups, in addition to interference with ADA by other inflammatory aetiologies. Nevertheless, ADA is a widely used biomarker for screening TPE due to its simplicity, rapidity, and low financial cost, but the above results proved that overreliance on ADA differentiation may lead to missed diagnosis/misdiagnosis in clinical settings, especially in the indeterminate range. In addition, the PF T-SPOT assay could fill this gap.

In addition to age, we screened two additional high-risk factors that were significantly related to TPE, sex and BMI. There were 5 non-TPE patients scoring 11 (simultaneously having three high-risk factors: age < 45 yrs., male sex and BMI < 22), which directly demonstrated that these clinical characteristics could be an effective reference index for discerning TPE from other PEs. We often defined these patients as the population with a high incidence of TPE, also called the typical population. However, it is notable that the utility of ADA fluctuated distinctly by stratified analysis, and if it did not satisfy any one condition (defined as the unconventional population), it was inferior to the T-SPOT assay. The unconventional population is frequently the focal point and has difficulties in diagnosis; as a result, the PF T-SPOT assay can provide powerful identification evidence for these patients.

There are several limitations in the current study. First, the entire study was performed in a single centre that specialized in TB. The geography, relative single control composition and aetiological attribution error and/or bias were incalculable. Second, we obtained the optimal cutoff for the PF T-SPOT.TB assay from ROC analysis in this training cohort, and its definite accuracy would need further validation. Finally, 38.1% of the clinically diagnosed patients lacked the aetiological basis due to objective factors such as no sputum or unsatisfactory sputum with the detection standard, which may bias the sputum detection rate.

## Conclusion

In conclusion, neither ADA nor IGRA can be defined as definite proof of ruling in or out TPE, but they can be used as powerful reference tools for TPE diagnosis. The overall accuracy of the PF T-SPOT assay was equal to that of ADA for diagnosing TPE in our study. In addition, the PF T-SPOT assay can effectively discriminate TPE patients with low ADA and is extremely superior to ADA in unconventional TPE patients (age ≥ 45 yrs., female or BMI ≥ 22), indicating that the applied population is wider than that of ADA. The PF T-SPOT assay is an excellent choice to supplement ADA to diagnose TPE.

## Data Availability

The datasets analysed during the current study are not publicly available but are available from the corresponding author on reasonable request.

## Abbreviations

AFB: Acid-fast bacilli
ADA: Adenosine deaminase
AUC: Area under the curve
BMI: Body mass index
CRS: Composite reference standard
CI: Confidence interval
CFP-10: Culture filtrate protein-10
DOR: Diagnostic odds ratio
ESAT-6: Early secretory antigenic target-6
ELISPOT: Enzyme-linked immunosorbent spot
ECDC: European Centre for Disease Prevention and Control
IGRA: Interferon gamma release assay
IFN-γ: Interferon gamma
IQR: Interquartile range
LDH: Lactate dehydrogenase
+LR: Positive likelihood ratio
-LR: Negative likelihood ratio
MTB: *Mycobacterium tuberculosis*
NPV: Negative predictive value
OR: Odds ratio
PB: Peripheral blood
PBMCs: Peripheral blood mononuclear cells
PE: Pleural effusion
PFMCs: Pleural fluid mononuclear cells
PF: Pleural fluid
PPV: Positive predictive value
ROC: Receiver operating characteristic
SFCs: Spot-forming cells
TB: Tuberculosis
TPE: Tuberculous pleural effusion
WHO: World Health Organization
